# *In Vitro* Functional Characterization of GET73 as Possible Negative Allosteric Modulator of Metabotropic Glutamate Receptor 5

**DOI:** 10.3389/fphar.2018.00327

**Published:** 2018-04-05

**Authors:** Sarah Beggiato, Andrea C. Borelli, Maria C. Tomasini, M. Paola Castelli, Nicholas Pintori, Roberto Cacciaglia, Antonella Loche, Luca Ferraro

**Affiliations:** ^1^Department of Life Sciences and Biotechnology, University of Ferrara, Ferrara, Italy; ^2^IRET Foundation, Bologna, Italy; ^3^Department of Biomedical Sciences, University of Cagliari, Cagliari, Italy; ^4^Center of Excellence “Neurobiology of Addiction”, University of Cagliari, Cagliari, Italy; ^5^Laboratorio Farmaceutico CT Srl, Sanremo, Italy; ^6^LTTA Centre, University of Ferrara, Ferrara, Italy

**Keywords:** intracellular calcium, CREB, Inositol trisphosphate, alcohol dependence, mGluR5

## Abstract

The present study was aimed to further characterize the pharmacological profile of N-[4-(trifluoromethyl) benzyl]-4-methoxybutyramide (GET73), a putative negative allosteric modulator (NAM) of metabotropic glutamate subtype 5 receptor (mGluR5) under development as a novel medication for the treatment of alcohol dependence. This aim has been accomplished by means of a series of *in vitro* functional assays. These assays include the measure of several down-stream signaling [intracellular Ca^++^ levels, inositol phosphate (IP) formation and CREB phosphorylation (pCREB)] which are generally affected by mGluR5 ligands. In particular, GET73 (0.1 nM–10 μM) was explored for its ability to displace the concentration-response curve of some mGluR5 agonists/probes (glutamate, L-quisqualate, CHPG) in different native preparations. GET73 produced a rightward shift of concentration-response curves of glutamate- and CHPG-induced intracellular Ca^++^ levels in primary cultures of rat cortical astrocytes. The compound also induced a rightward shift of concentration response curve of glutamate- and L-quisqualate-induced increase in IP turnover in rat hippocampus slices, along with a reduction of CHPG (10 mM)-induced increase in IP formation. Moreover, GET73 produced a rightward shift of concentration-response curve of glutamate-, CHPG- and L-quisqualate-induced pCREB levels in rat cerebral cortex neurons. Although the engagement of other targets cannot be definitively ruled out, these data support the view that GET73 acts as an mGluR5 NAM and support the significance of further investigating the possible mechanism of action of the compound.

## Introduction

Alcohol dependence is a chronic relapsing disorder, which continues to be a concerning health and socio-economic issue worldwide (World Health Organization [WHO], 2014). Several national and international health Organizations activated programs aimed at monitoring the burden related to alcohol dependence and at promoting the development of new medical interventions, in order to effectively treat this disorder. In fact, possibly due to the neurobiological complexity and the clinical heterogeneity of alcohol dependence, the effects of the drugs currently approved in the United States for alcohol dependence treatment (i.e., acamprosate, naltrexone, nalmefene and disulfiram) are modest/moderate, both in terms of number needed to treat and effects size (Spanagel, 2009; Swift and Aston, 2015; Goh and Morgan, 2017; Soyka and Müller, 2017). Consequently, there is an urgent medical need for the identification of new pharmacological targets and for the development of new and more effective medications (Leggio et al., 2010; Edwards et al., 2011; Soyka and Lieb, 2015; Mason, 2017; Soyka and Müller, 2017). The development of N-[4-(trifluoromethyl) benzyl]-4-methoxybutyramide (GET73) falls within this context. In preclinical studies this compound has shown the ability to reduce alcohol intake along with anxiolytic-like properties (Loche et al., 2012; Ferraro et al., 2013). Its possible mechanism of action has been related to the modulation of glutamate neurotransmission through the metabotropic glutamate subtype 5 receptor (mGluR5) (Ferraro et al., 2011, 2013; Beggiato et al., 2013), a promising target for the development of pharmacological alcohol dependence treatments (Olive, 2009; Duncan and Lawrence, 2012; Holmes et al., 2013; Goodwani et al., 2017), and for many other psychiatric conditions, such as anxiety and depressive states (Tatarczynska et al., 2001). The results of several Phase 1 clinical studies indicate that GET73 is safe and well-tolerated both in healthy volunteers (Haass-Koffler et al., 2017a,b), and in alcohol-dependent patients (ongoing study NCT01842503)^1^.

The mechanism of action of GET73 is still under study. This compound did not show affinity for a series of biological targets involved in drug addiction and alcohol dependence, including dopamine, serotonin, GABA, ionotropic glutamate receptors, along with dopamine and serotonin reuptake systems (Loche et al., 2012); despite this negligible binding profile, the compound affected GABA and glutamate neurotransmission in the rat hippocampus (Ferraro et al., 2011; Beggiato et al., 2013). Based on these data, additional studies were addressed to explore the possibility that GET73 could modulate metabotropic glutamate receptor (mGluR) functions, and specifically mGluR5. To this purpose, experiments were performed to evaluate the interaction between GET73 and two mGluR5 ligands, the orthosteric mGluR5 agonist (R,S)-2-chloro-5-hydroxyphenylglycine (CHPG), and the mGluR5 NAM 2-methyl-6-(phenylethynyl) pyridine hydrochloride (MPEP). The results of both *in vitro* and *in vivo* microdialysis studies carried out in the rat hippocampus, suggested that GET73 might exert a double negative/positive allosteric modulation at mGluR5. Specifically, low nM concentrations exerted a negative modulation at mGluR5, and a possible amplification of MPEP-induced effects. On the other hand, the GET73-induced increase in glutamate and GABA signaling, exerted at higher μM concentrations, were counteracted by MPEP, suggesting that GET73 might also exert a positive modulation at mGluR5, at least in the model employed for those studies (Ferraro et al., 2011, 2013; Beggiato et al., 2013). However, the above studies only indirectly suggested the existence of a possible interaction between GET73 and mGluR5.

The present study was aimed at further investigating the interactions between GET73 and mGluR5 by means of a series of *in vitro* functional assays. Briefly, GET73 was explored for its ability to displace the concentration-response curve of some mGluR5 agonists/probes (glutamate, L-quisqualate, CHPG) on different intracellular signaling molecules/pathways (intracellular Ca^++^ levels, IP formation and pCREB). GET73 effects were explored in different native systems, including the hippocampus, the brain area extensively explored for the neuropharmacological characterization of the compound.

## Materials and Methods

### Chemicals

Glutamate, the selective mGluR5 agonist *(RS)*-2-Chloro-5-hydroxyphenylglycine (CHPG) and the AMPA/group I mGluR agonist (L)-(+)-α-Amino-3,5-dioxo-1,2,4-oxadiazolidine-2-propanoic acid (L-quisqualate) were purchased from Tocris (Ballwin, MO, United States). GET73 was synthetized by Laboratorio CT (Sanremo, Italy).

### Animals

Adult male Sprague Dawley rats (Charles-River, Milan, Italy) were used in this study. Animals were housed 4 per cage at a temperature of 22°C and 60% humidity under a 12-h light/dark cycle (lights on from 7.00 am). Tap water and standard laboratory rodent chow (Mucedola, Settimo Milanese, Italy) were provided *ad libitum* in the home cage. The experimental protocols performed in this study were in accordance with the new European Communities Council Directive of September 2010 (2010/63/EU) a revision of the Directive 86/609/EEC and were approved by the Italian Ministry of Health and by the Ethical Committee of the University of Ferrara (D.M. n° 104/2017).

### Calcium Mobilization Experiments

The experiments have been performed on primary cultures of rat cortical astrocytes. These cells express mGlu5Rs (Zhang et al., 2005).

#### Primary Cultures of Rat Cortical Astrocyte Preparation

Primary cultures of cerebral cortical astrocytes were obtained from newborn rats (1 or 2 days old). Briefly, cerebral cortices were removed and dissociated by mild trypsinization at 37°C, followed by mechanical trituration to obtain single cells. Cells were suspended in the culture medium [Gibco^®^ DMEM, 5% inactivated fetal bovine serum (Thermo-Fisher Scientific, Waltham, MA, United States), 100 IU/ml penicillin, and 100 μg/ml streptomycin (all from Sigma-Aldrich, Milan, Italy)] and then seeded in 75-cm^2^ flasks at a density of 3 × 10^6^ cells/flask. The cells were incubated at 37°C in a humidified atmosphere, 5% CO_2_/95% air. The culture medium was replaced after 24 h and again twice weekly until astrocytes were grown to form a monolayer firmly attached to the bottom of the flask (12 or 14 days after dissection). At cell confluence, flasks were vigorously shaken to separate astrocytes (which remained adherent in the bottom of the flasks) from microglia and oligodendrocytes (which floated on the supernatant) (Tomasini et al., 2015). Collected astrocytes were counted and then plated in poly-L-lysine at a density of 50,000 cells/well or 96-well plates black, clear bottom plates (Corning^®^; Sigma-Aldrich, Milan, Italy).

#### Calcium Mobilization Measurement

After 24 h of incubation, the cells were loaded with Hank’s Balanced Salt Solution (HBSS) supplemented with 2.5 mM probenecid (Sigma-Aldrich, Milan, Italy), 3 μM of the calcium sensitive fluorescent dye Fluo-4 AM (Thermo-Fisher Scientific, Waltham, MA, United States), 0.01% pluronic acid and 20 mM HEPES (pH 7.4; Sigma-Aldrich, Milan, Italy) for 30 min at 37°C. Afterwards the loading solution was aspirated, a washing step with 100 μl/well of HBSS, HEPES (20 mM, pH 7.4), 2.5 mM probenecid and 500 μM Brilliant Black (Sigma-Aldrich, Milan, Italy) was carried out. Subsequently 100 μl/well of the same buffer were added for 10 min. Concentrated solutions of ligands (i.e., glutamate, L-quisqualate, CHPG and GET73) were freshly prepared and serial dilutions were made in HBSS/HEPES (20 mM) buffer (containing 0.02% BSA fraction V). After placing cell culture and compound plates into the FlexStation II (Molecular Devices, Sunnyvale, CA, United States), the on-line additions were carried out in a volume of 50 μl /well and fluorescence changes were continuously measured for 2 min at 37°C. The calcium peak level has been then selected to evaluate the effects of treatments.

### Inositol Phosphate Turnover Experiments

The experiments have been performed on rat hippocampus slices. This preparation expresses mGlu5Rs (Zhang et al., 2005; Purgert et al., 2014).

#### Rat Hippocampal Slices

On the day of the experiment, the animals were euthanized, their brain promptly isolated and 400 μm thick slices (15–20 mg each) were obtained from both the left and right hippocampi, by using a McIlwain tissue Chopper fresh tissue. The tissue was then allowed to equilibrate for 20 min at room temperature in Krebs’ solution (composition in mM: NaCl 118; KC1 4.4; CaC1_2_ 1.2; MgS0_4_ 1.2; KH_2_P0_4_ 1.2; NaHC0_3_ 25; glucose 10) and gassed with a mixture of 95% O_2_ plus 5% CO_2_ (Ferraro et al., 2011).

#### Measurements of Inositol Phosphate Turnover in Rat Hippocampus Slices

Slices were placed in test tubes containing 0.3 ml of oxygenated Krebs solution with myo-[^3^H]inositol 0.3 μM (NEN, Boston, MA, United States). After 30 min of incubation, glutamate receptor agonists (i.e., glutamate, L-quisqualate, CHPG) were added and the incubation was prolonged for a further 30 min period (Morari et al., 1994). The concentration-response curves of glutamate receptor agonists were assessed in the absence or in the presence of different concentrations of GET73, incubated 15 min before the addition of glutamate receptor agonists. The reaction was stopped by washing each slice in ice cold Krebs solution containing LiCl 10 mM and by plunging it in 0.94 ml of chloroform-methanol (1:2 v/v). After 10 min, 310 μl of chloroform and 310 μl of water were added and the tubes were shaken for 20 min. The separation of [^3^H]inositol phosphates was performed by ion-exchange chromatography on AG 1-X8 resin, 200–400 mesh (Bio-Rad, Hercules, CA, United States), using different elution solutions. The collected IP_3_-containing fraction was mixed with Safety-Solve cocktail (RPI, Mount Prospect, IL, United States) and the radioactivity was measured by scintillation counting.

### pCREB Experiments

The experiments have been performed on primary cultures of rat cortical neurons. These cells express mGlu5Rs (Koga et al., 2010).

#### Primary Cultures of Rat Cortical Neuron Preparation

Primary cultures of cerebral cortical neurons were prepared from 1-day-old rats. Cortices free of meninges were dissociated in 0.025% (w/v) trypsin at 37°C followed by mechanical repeated gentle pipetting through wide- and narrow-bore fire-polished Pasteur pipettes in culture medium [Neurobasal medium (Gibco, Grand Island, NY, United States) supplemented with 0.1 mM glutamine (Sigma-Aldrich, Milan, Italy), 10 μg/ml gentamicin (Sigma-Aldrich, Milan, Italy) and 2% B-27^®^ Supplement (50X), serum free (Gibco, Grand Island, NY, United States)]. Cells were counted and then plated on poly-L-lysine (5 μg/ml)-coated multiwells. Cytosine arabinoside (10 μM; Sigma-Aldrich, Milan, Italy) was added within 24 h of plating to prevent glial cell proliferation. After 8 days of *in vitro* incubation (days *in vitro*: DIV), cultures were used for experiments.

#### pCREB Measurement

pCREB measurement was performed with the pCREB (Ser133) Assay kit (PerkinElmer Italy, Milan). Briefly, cortical neurons were seeded in 96 well tissue culture plates at a density of 4,000 cells/well and incubated at 37°C overnight. Thereafter, the concentration-response curves of glutamate receptor agonists (i.e., glutamate, L-quisqualate, CHPG) were assessed in the absence or in the presence of GET73. Briefly, the cells were incubated for 1 h with the glutamate receptor agonists, lysed with the freshly prepared lysis buffer. When required, GET73 was applied at different concentrations 15 min before the addition of glutamate receptor agonists. The lysates were transferred to a 96-well ½AreaPlate^TM^ for the assay. The Acceptor Mix was added for 1 h at room temperature and the Donor mix was added to wells under subdue light and incubate for 1 h at room temperature. At the end of this period, raw “AlphaScreen Signal counts” were measured by an Alpha Technology^®^-compatible plate reader, using standard AlphaLISA settings (excitation 680 nm, emission 615 nm).

### Data Analysis

EC_50_ values were calculated using non-linear regression [curve fit, log(agonist) vs. response, variable slope, four parameters]. Statistical analysis (*F*-test) has been performed by using GraphPad Prism 6.0 (GraphPad Software Inc., San Diego, CA, United States). Statistical differences between group means for EC_50_ values were also assessed by ANOVA followed by Tukey’s multiple comparisons test. Data are reported as mean ± SEM of three independent experiments. A *p*-value < 0.05 was considered statistically significant.

## Results

### Calcium Mobilization Experiments

#### Agonistic Effects

**FIGURE 1:**
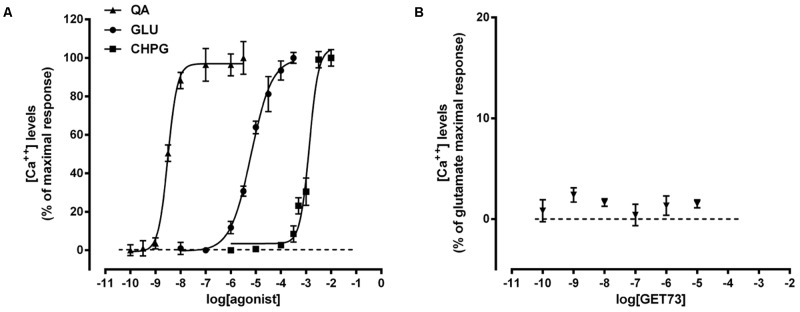
**(A)** Glutamate receptor agonist concentration-response curves in primary cultures of rat cortical astrocytes. The effects of the treatments on intracellular calcium levels are expressed as % of maximal response over the basal values. The EC_50_ values were: L-quisqualate (QA) = 3.1 ± 0.36 nM; glutamate (GLU) = 6.5 ± 0.44 μM; *(RS)-*2-Chloro-5-hydroxyphenylglycine (CHPG) = 1.3 ± 0.09 mM. Concentration-response curves were generate using non-linear regression [curve fit, log(agonist) vs. response, variable slope, four parameters]. **(B)** Effects of GET73 (0.1 nM–10 μM) on intracellular calcium levels in primary cultures of rat cortical astrocytes. The effects of the treatment are expressed as % of glutamate maximal response over the basal values. Each point represents the mean ± SEM (*n* = 3).

As expected, glutamate, L-quisqualate and CHPG concentration-dependently induced calcium transient in primary cultures of rat cortical astrocytes. The profiles of the concentration-response curves were in line with the known EC_50_ values of the compounds (**Figure 1A** and **Table 1**; Acher, 2006). On the contrary, GET73 (0.1 nM–10 μM) was ineffective (**Figure 1B**).

**Table 1:** EC_50_ values obtained with glutamate, CHPG or L-quisqualate in the absence or in the presence of GET73 (0.1 nM–10 μM) by evaluating intracellular calcium levels in primary cultures of rat cortical astrocytes.

Agonist	GET73 (concentration)	EC_50_
Glutamate	0	6.5 ± 0.44 μM
	0.1 nM	9.0 ± 0.51 μM
	1 nM	7.8 ± 0.36 μM
	10 nM	40 ± 2.8 μM**°°
	100 nM	21 ± 1.4 μM**
	1 μM	24 ± 1.7 μM**
	10 μM	16 ± 0.54 μM**
CHPG	0	1.3 ± 0.09 mM
	0.1 nM	2.0 ± 0.13 mM*°
	1 nM	3.8 ± 0.28 mM**°°^§^
	10 nM	3.6 ± 0.37 mM**°°^§^
	100 nM	2.7 ± 0.22 mM**
	1 μM	2.8 ± 0.18 mM**
	10 μM	2.6 ± 0.24 mM**
L-quisqualate	0	3.1 ± 0.36 nM
	0.1 nM	2.9 ± 0.45 nM
	1 nM	3.5 ± 0.69 nM
	10 nM	3.3 ± 0.55 nM
	100 nM	3.2 ± 0.62 nM
	1 μM	3.6 ± 0.54 nM
	10 μM	3.6 ± 0.63 nM

Results are expressed as mean ± SEM of 3 independent experiments. EC_50_ values were calculated using non-linear regression [curve fit, log(agonist) vs. response, variable slope, four parameters]. Statistical differences between group means for EC_50_ values were also assessed by ANOVA followed by Tukey’s multiple comparisons test. Glutamate = *p < 0.05, **p < 0.01, significantly different from the control (i.e., GET73 0 concentration), GET73 0.1 and 1 nM groups; °p < 0.05, °°p < 0.01, significantly different from the GET73 100 nM, 1 µM, and 10 µM groups; CHPG = *p < 0.05, **p < 0.01, significantly different from the control (i.e., GET73 0 concentration); °p < 0.05, °°p < 0.01, significantly different from the GET73 100 nM, 1 µM, and 10 µM groups; ^§^p < 0.05 significantly different from the GET73 0.1 nM group.

#### Effects of GET73 on Glutamate Concentration-Response Curve in Primary Cultures of Rat Cortical Astrocytes

**FIGURE 2:**
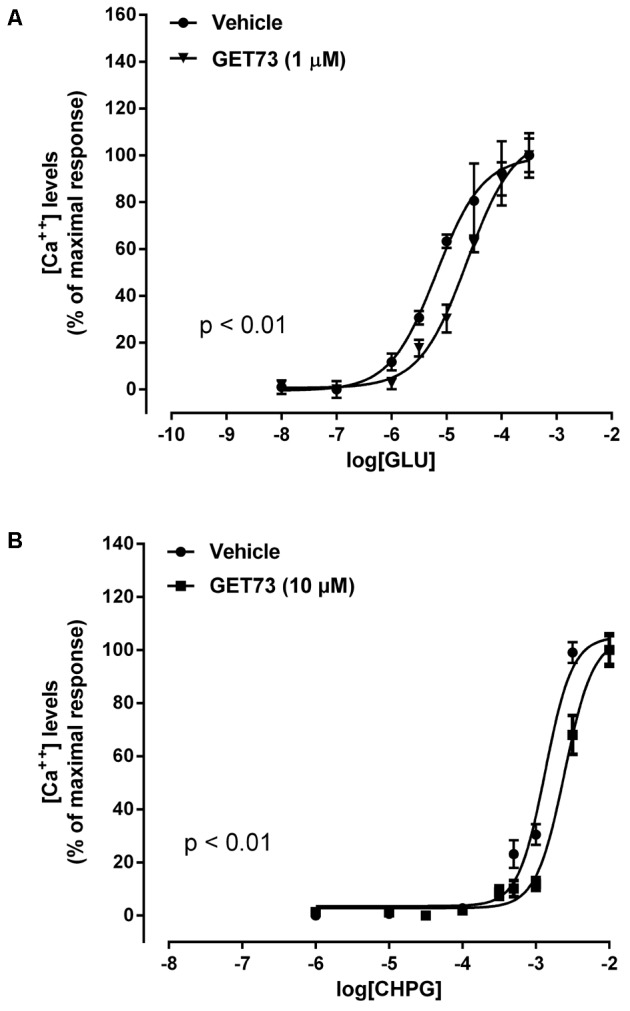
Effects of GET73 on glutamate (GLU; **A**) and *(RS)-*2-Chloro-5-hydroxyphenylglycine (CHPG; **B**) concentration-dependent increase in intracellular calcium levels in primary cultures of rat cortical astrocytes. The effects of the treatments on intracellular calcium levels are expressed as % of maximal response over the basal values. Each point represents the mean ± SEM (*n* = 3). Concentration-response curves were generate using non-linear regression [curve fit, log(agonist) vs. response, variable slope, four parameters].

The concentration-response curves for glutamate were performed either in the absence or presence of GET73 (see **Figure 2A**, GET73 1 μM, as an example); GET73 (10 nM–10 μM) induced a significant rightward shift of glutamate concentration-response curve in primary cultures of rat cortical astrocytes (Supplementary Figure 1), thus increasing the glutamate EC_50_ value (**Table 1**) without a substantial change in the maximal response. The analysis of the apparent EC_50_ values revealed a bell-shaped profile; in fact the maximum effect was observed at 10 nM, while the effects of higher concentrations (100 nM, 1 μM, 10 μM) were similar but lower than the maximum one. At lower concentrations (0.1 and 1 nM), GET73 was ineffective.

#### Effects of GET73 on CHPG Concentration-Response Curve in Rat Cortical Astrocytes

The concentration-response curves for CHPG were performed either in the absence or presence of GET73 (see **Figure 2B**, GET73 10 μM, as an example). GET73, at all concentrations tested (0.1 nM–10 μM), induced a significant rightward shift of the CHPG concentration-response curve in primary cultures of rat cortical astrocytes (Supplementary Figure 2 and **Table 1** for EC_50_ values), without a substantial change in the maximal response. The analysis of the apparent EC_50_ values revealed a bell-shaped profile; in fact the maximum effect was observed at 1 and 10 nM, while at higher concentrations (100 nM, 1 μM, 10 μM) the compound induced similar effects that were significantly higher than that observed at 0.1 nM concentration, but lower than the maximum one.

#### Effects of GET73 on L-quisqualate Concentration-Response Curve in Rat Cortical Astrocytes

The concentration-response curves for L-quisqualate were performed either in the absence or presence of GET73. At all concentrations tested (0.1 nM–10 μM) GET73 failed to significantly modify the L-quisqualate concentration-response curve in primary cultures of rat cortical astrocytes (Supplementary Figure 3 and **Table 1**).

### Phosphatidylinositol Turnover in Rat Hippocampus Slices

#### Agonistic Effects

**FIGURE 3:**
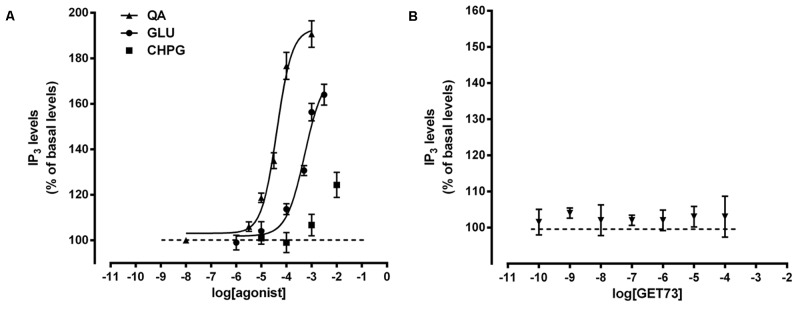
**(A)** Concentration-dependent increases of phosphatidylinositol turnover (IP_3_) induced by glutamate receptor agonist in rat hippocampus slices. The EC_50_ values were: L-quisqualate (QA) = 40 ± 2.37 μM; glutamate (GLU) = 0.68 ± 0.06 mM. Concentration-response curves were generate using non-linear regression [curve fit, log(agonist) vs. response, variable slope, four parameters]. CHPG = *(RS)-*2-Chloro-5-hydroxyphenylglycine. **(B)** Effects of GET73 (0.1 nM–10 μM) of phosphatidylinositol turnover (IP_3_) in rat hippocampus slices. Results are expressed as % of basal levels, always assessed in parallel. Each point represents the mean ± SEM (*n* = 3).

As expected, glutamate and L-quisqualate concentration-dependently increased phosphatidylinositol turnover in rat hippocampal slices (**Figure 3A**). Under the present experimental conditions, the resulting EC_50_ values were higher than the known EC_50_ values of the compounds (Acher, 2006) as well as than those observed by evaluating the effects of the compounds on intracellular calcium (**Figure 1A**). On the contrary, CHPG (0.01–10 mM) significantly increased phosphatidylinositol turnover only at the higher concentration tested (127 ± 4% of basal levels; *p* < 0.05; **Figure 3A**). GET73, at all concentrations tested (0.1 nM–100 μM), failed to modify phosphatidylinositol turnover in rat hippocampal slices (**Figure 3B**).

#### Effects of GET73 on Glutamate Concentration-Response Curve in Rat Hippocampus Slices

**FIGURE 4:**
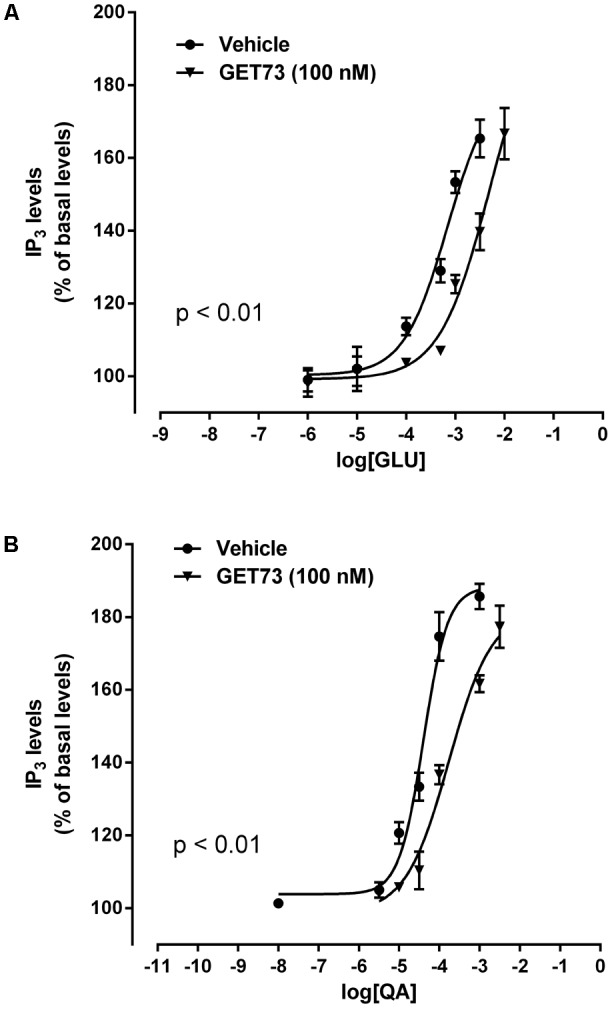
Effects of GET73 (100 nM) on glutamate (GLU; **A**) and L-quisqualate (QA; **B**) concentration-dependent increase of phosphatidylinositol turnover (IP_3_) in rat hippocampus slices. Results are expressed as % of basal levels, always assessed in parallel. Each point represents the mean ± SEM (*n* = 3). Concentration-response curves were generate using non-linear regression [curve fit, log(agonist) vs. response, variable slope, four parameters].

The concentration-response curves for glutamate were performed either in the absence or presence of GET73 (see **Figure 4A**, GET73 100 nM, as an example). At the concentrations of 100 nM–10 μM GET73 induced a significant rightward shift of the glutamate concentration-response curve in rat hippocampus slices (Supplementary Figure 4) thus increasing the EC_50_ value (**Table 2**). The analysis of the apparent EC_50_ values revealed a bell-shaped profile; in fact the maximum effect was observed at 100 nM, while the effects of the lower 10 nM concentration and the higher 1 and 10 μM concentrations were similar, but lower than the maximum one. At lower concentrations (0.1 and 1 nM), GET73 was ineffective.

**Table 2:** EC_50_ values obtained with glutamate or L-quisqualate in the absence or in the presence of GET73 (0.1 nM–10 μM) by evaluating phosphatidylinositol turnover in rat hippocampus slices.

Agonist	GET73 (concentration)	EC_50_
Glutamate	0	0.68 ± 0.06 mM
	0.1 nM	0.76 ± 0.07 mM
	1 nM	0.90 ± 0.07 mM
	10 nM	3.0 ± 0.24 mM**
	100 nM	5.0 ± 0.29 mM**°°
	1 μM	2.3 ± 0.33 mM**
	10 μM	3.2 ± 0.42 mM**
L-quisqualate	0	40 ± 2.37 µM
	0.1 nM	33 ± 1.57 µM
	1 nM	40 ± 1.44 µM
	10 nM	110 ± 9.5 µM**
	100 nM	200 ± 11.1 µM**^§^
	1 μM	320 ± 15.7 µM**°°^§^
	10 μM	200 ± 12.6 µM**^§^

EC_50_ values were calculated using non-linear regression [curve fit, log(agonist) vs. response, variable slope, four parameters]. Statistical differences between group means for EC_50_ values were also assessed by ANOVA followed by Tukey’s multiple comparisons test. Glutamate = **p < 0.01, significantly different from the control (i.e., GET73 0 concentration), GET73 0.1 and 1 nM groups; °°p < 0.01, significantly different from the GET73 10 nM, 1 µM and 10 µM groups; L-quisqualate = **p < 0.01, significantly different from the control (i.e., GET73 0 concentration), GET73 0.1 and 1 nM groups; °°p < 0.01, significantly different from the GET73 100 nM and 10 µM groups; ^§^ p < 0.05 significantly different from the GET73 10 nM group.

#### Effects of GET73 on L-quisqualate Concentration-Response Curve in Rat Hippocampus Slices

The concentration-response curves for L-quisqualate were performed either in the absence or presence of GET73 (see **Figure 4B**, GET73 100 nM, as an example). At the concentration of 10 nM–10 μM GET73 induced a significant rightward shift of the L-quisqualate concentration-response curve in rat hippocampus slices (Supplementary Figure 5 and **Table 2** for EC_50_ values), without substantially changing the maximal response. The analysis of the apparent EC_50_ values revealed a bell-shaped profile; in fact the effect of the compound concentration-dependently increase in the concentration range of 10 nM–1 μM, while at the higher concentration tested (10 μM) the compound induced an increase in glutamate EC_50_ value which was lower to that induced by GET73 1 μM, and comparable to GET73 100 nM- provoked effect. Finally, at lower concentrations (0.1 and 1 nM), GET73 was ineffective.

#### Effects of GET73 on CHPG-Induced Alterations of IP_3_ Levels in Rat Hippocampus Slices

**FIGURE 5:**
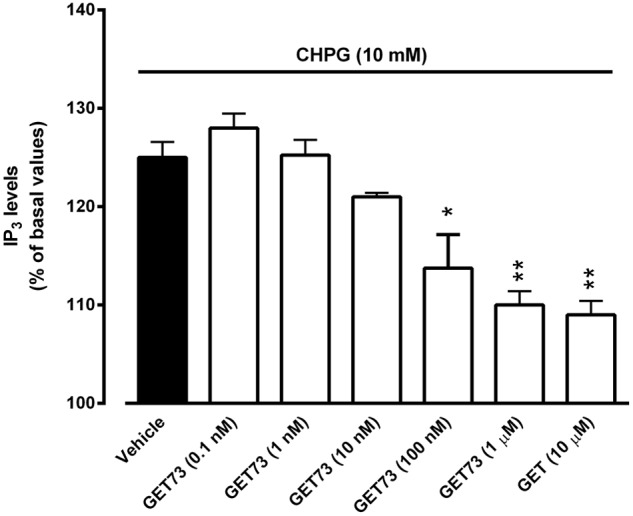
Effects of GET73 (0.1 nM–10 μM) on CHPG (10 mM)-induced increase of phosphatidylinositol turnover (IP_3_) in rat hippocampus slices. Results are expressed as % of basal levels and each histogram represents the mean ± SEM of 3 experiments. *p < 0.05; **p < 0.01 significantly different from vehicle group according to ANOVA followed by Tukey test for multiple comparisons.

At the concentrations of 100 nM, 1 μM and 10 μM, GET73 induced a significant reduction of CHPG (10 mM)-induced increase in phosphatidylinositol turnover in rat hippocampus slices (**Figure 5**). At lower concentrations (0.1–10 nM), GET73 was ineffective.

### CREB Phosphorylation Experiments

#### Agonistic Effects

Preliminary experiments indicated that in primary cultures of rat cortical astrocytes the glutamate-, L-quisqualate- or CHPG-induced pCREB levels were very low (*data not shown*). This is in line with previous published results (Mao and Wang, 2002, 2003). Thus, the experiments on pCREB levels were performed in cerebral cortex neurons.

**FIGURE 6:**
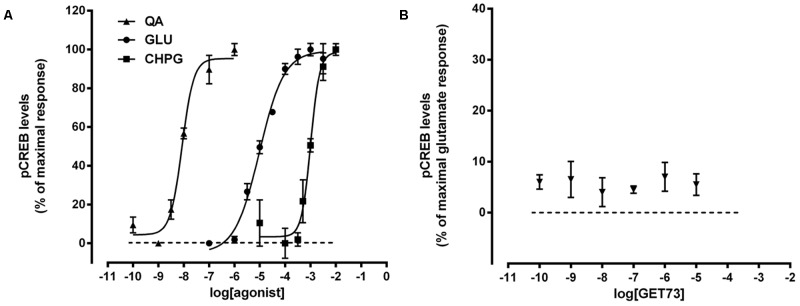
**(A)** Concentration-dependent increases in phosphorylated CREB (pCREB) levels induced by glutamate receptor agonist in primary cultures of rat cortical neurons. The effects of the treatments on pCREB levels are expressed as % of maximal response over the basal values. The EC_50_ values were: L-quisqualate (QA) = 8.8 ± 0.68 nM; glutamate (GLU) = 9.7 ± 0.78 μM; *(RS)-*2-Chloro-5-hydroxyphenylglycine (CHPG) = 1.0 ± 0.09 mM. Concentration-response curves were generate using non-linear regression [curve fit, log(agonist) vs. response, variable slope, four parameters]. **(B)** Effects of GET73 (0.1 nM–10 μM) on phosphorylated CREB (pCREB) levels in primary cultures of rat cortical neurons. The effects of the treatment are expressed as % of glutamate maximal response over the basal values. Each point represents the mean ± SEM (*n* = 3).

As expected, glutamate, L-quisqualate and CHPG caused concentration-dependent increases in pCREB levels (**Figure 6A**). The profiles of the concentration-response curves were in line with the known EC_50_ values of the compounds and comparable to those observed by evaluating the effects of the glutamate receptor agonist on intracellular calcium levels (**Figure 1A**; Acher, 2006). On the contrary, GET73 (0.1 nM–10 μM) was ineffective (**Figure 6B**).

#### Effects of GET73 on Glutamate Concentration-Response Curve in Primary Cultures of Rat Cortical Neurons

**FIGURE 7:**
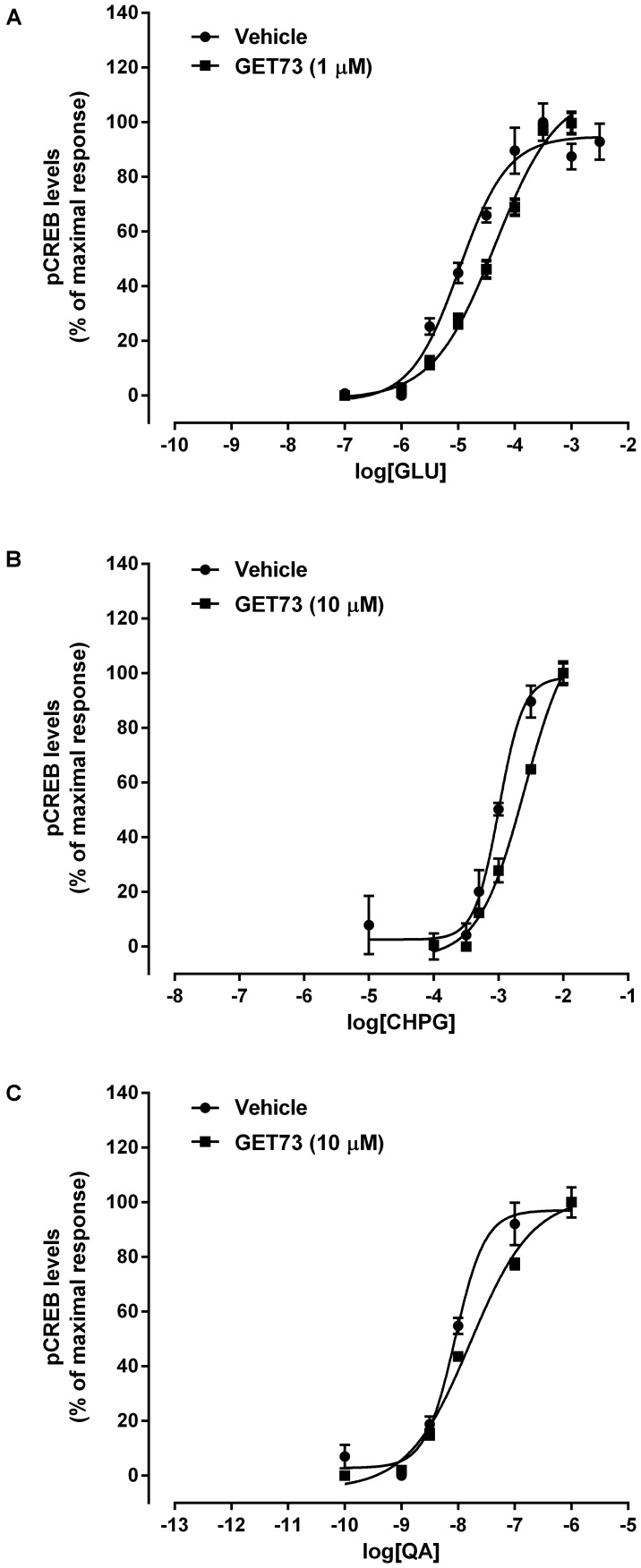
Effects of GET73 (1 or 10μM) on glutamate (GLU; **A**), *(RS)-*2-Chloro-5-hydroxyphenylglycine (CHPG; **B**) and L-quisqualate (QA; **C**) concentration-dependent increase in phosphorylated CREB (pCREB) levels in primary cultures of rat cortical neurons. The effects of the treatments are expressed as % of maximal response over the basal values. Each point represents the mean ± SEM (*n* = 3). Concentration-response curves were generate using non-linear regression [curve fit, log(agonist) vs. response, variable slope, four parameters].

The concentration-response curves for glutamate were performed either in the absence or presence of GET73 (see **Figure 7A**, GET73 1 μM, as an example). GET73 (10 nM–10 μM) induced a significant rightward shift of the glutamate concentration-response curve in primary cultures of rat cortical neurons, thus increasing the EC_50_ value (Supplementary Figure 6 and **Table 3** for EC_50_ values) without substantially changing the maximal response. The analysis of the apparent EC_50_ values revealed a bell-shaped profile; in fact the effect of the compound concentration-dependently increase in the concentration range of 10 nM–1 μM, while at the higher concentration tested (10 μM) the compound induced an increase in glutamate EC_50_ value which was lower to that induced by GET73 1 μM, and comparable to GET73 100 nM- provoked effect. Finally, at lower concentrations (0.1 and 1 nM), the compound was ineffective.

**Table 3:** EC_50_ values obtained with glutamate, CHPG or L-quisqualate in the absence or in the presence of GET73 (0.1 nM–10 μM) by evaluating pCREB levels in cerebral cortex neurons.

Agonist	GET73 (concentration)	EC_50_
Glutamate	0	9.7 ± 0.78 µM
	0.1 nM	10 ± 0.88 µM
	1 nM	10 ± 0.64 µM
	10 nM	25 ± 1.41 µM**
	100 nM	35 ± 1.53 µM**^§^
	1 μM	45 ± 1.39 µM**°°^§^
	10 μM	31 ± 1.34 µM**
CHPG	0	1.0 ± 0.09 mM
	0.1 nM	1.0 ± 0.08 mM
	1 nM	0.9 ± 0.04 mM
	10 nM	1.4 ± 0.12 mM
	100 nM	5.0 ± 0.29 mM**°°^§§^
	1 μM	3.6 ± 0.34 mM**^§^
	10 μM	2.6 ± 0.28 mM**
L-quisqualate	0	8.8 ± 0.68 nM
	0.1 nM	9.0 ± 0.91 nM
	1 nM	7.4 ± 0.66 nM
	10 nM	8.5 ± 0.52 nM
	100 nM	11 ± 0.96 nM
	1 μM	22 ± 1.72 nM**°
	10 μM	16 ± 0.44 nM**

EC_50_ values were calculated using non-linear regression [curve fit, log(agonist) vs. response, variable slope, four parameters]. Statistical differences between group means for EC_50_ values were also assessed by ANOVA followed by Tukey’s multiple comparisons test. Glutamate = **p < 0.01, significantly different from the control (i.e., GET73 0 concentration), GET73 0.1 and 1 nM groups; °°p < 0.01, significantly different from the GET73 100 nM and 10 µM groups; ^§^ p < 0.05 significantly different from the GET73 10 nM group; CHPG = **p < 0.01, significantly different from the control (i.e., GET73 0 concentration), GET73 0.1, 1 nM and 10 nM groups; °°p < 0.01, significantly different from the GET73 1 µM group; ^§^ p < 0.05, ^§§^ p < 0.01 significantly different from the GET73 10 nM group; L-quisqualate = **p < 0.01, significantly different from the control (i.e., GET73 0 concentration), GET73 0.1, 1, 10, and 100 nM groups; °p < 0.05, significantly different from the GET73 10 µM group.

#### Effects of GET73 on CHPG Concentration-Response Curve in Primary Cultures of Rat Cortical Neurons

The concentration-response curves for CHPG were performed either in the absence or presence of GET73 (see **Figure 7B**, GET73 10 μM, as an example). GET73 (100 nM–10 μM) induced a significant rightward shift of the CHPG concentration-response curve in primary cultures of rat cortical neurons (Supplementary Figure 7 and **Table 3** for EC_50_ values), without substantially changing the maximal response. The analysis of the apparent EC_50_ values revealed a bell-shaped profile; in fact the maximum effect was observed at 100 nM, while at higher concentrations (1 and 10 μM) the effects of the compound were progressively lower than the maximum one. At lower concentrations (0.1–10 nM), the compound was ineffective.

#### Effects of GET73 on L-quisqualate Concentration-Response Curve in Primary Cultures of Rat Cortical Neurons

The concentration-response curves for L-quisqualate were performed either in the absence or presence of GET73 (see **Figure 7C**, GET73 10 μM, as an example). GET73 at the concentrations of 1 and 10 μM induced a significant rightward shift of the L-quisqualate concentration-response curve in primary cultures of rat cortical neurons (Supplementary Figure 8 and **Table 3** for EC_50_ values), without substantially changing the maximal response. The analysis of the apparent EC_50_ values revealed a bell-shaped profile; in fact the maximum effect was observed at 1 μM, while at the higher concentration (10 μM) the effect of the compound was lower than the maximum one. At lower concentrations (0.1–100 nM), the compound was ineffective.

## Discussion

It has been formerly demonstrated that GET73 does not show affinity for a series of biological targets involved in drug addiction, including dopamine, serotonin, GABA, ionotropic glutamate receptors, along with dopamine and serotonin reuptake systems (Loche et al., 2012). Despite this negligible binding profile, the results of both *in vitro* (tissue slices) and *in vivo* microdialysis studies carried out in the rat hippocampus suggested that GET73 affects GABA and glutamate neurotransmission probably exerting a double negative/positive allosteric modulation at mGluR5. Specifically, low nanomolar GET73 concentrations seemed to exert a negative modulation at mGluR5, and a possible amplification of MPEP-induced effects. On the other hand, the effects of higher (i.e., μM) GET73 concentrations were counteracted by MPEP, suggesting that the compound might also exert a positive modulation at mGluR5 (Ferraro et al., 2011, 2013; Beggiato et al., 2013). Relevantly, the postulated ability of GET73 to negatively modulate mGluR5 may likewise explain, at least partially, its efficacy in reducing rat alcohol intake, as shown for several other compounds acting as NAM at mGluR5 (Olive, 2009; Duncan and Lawrence, 2012; Ferraro et al., 2013; Holmes et al., 2013; Mihov and Hasler, 2016; Goodwani et al., 2017). However, the above studies only indirectly suggested the existence of a possible interaction between GET73 and mGluR5. Thus, in the present study, functional evaluations have been performed to more directly proving that GET73 can modulate mGluR5 activity. The measurement of different parameters and the use of different *in vitro* preparations (i.e., primary cultures of cerebral cortex astrocytes or neurons, along with hippocampal slices) is justified by the evidence that agonism by PAMs and inhibition of the maximal response to glutamate by certain NAMs differed depending on receptor expression levels and the assays of receptor function (Kenakin, 2009; Gregory et al., 2012; Gregory and Conn, 2015). In particular, increasing evidence suggests that allosteric ligands can exhibit biased modulation of the orthosteric agonist, thus displaying phenotypic differences in their pharmacology on different intracellular pathways (Khoury et al., 2014; Rook et al., 2015; Nickols et al., 2016; Kenakin, 2017). Thus, the present study has been designed to cope with these critical features of allosteric modulation at GPCRs (i.e., probe-, pathway-, and system-dependence), also in order to reduce the risk of false negatives that always exists when only a single functional assay is performed (Conn et al., 2009; Gregory et al., 2012; Gregory and Conn, 2015). Taking into account these issues, the possible ability of GET73 to shift the concentration-response curves of different agonists/probes (i.e., glutamate, L-quisqualate, CHPG) on different intracellular pathways associated with mGluR5 activation (i.e., Ca^++^ levels, IP turnover and pCREB levels; Wang et al., 2007) and in different *in vitro* preparations (including the hippocampus, the brain area extensively explored for the neuropharmacological characterization of GET73), has been evaluated. Remarkably, the measure of mGluR5 agonist concentration-response curve displacement allow to detect both negative and positive modulation, providing a valuable approach for the characterization of a compound, such as GET73, that might exert either a negative or a positive modulation, depending on its concentration (Ferraro et al., 2011, 2013; Beggiato et al., 2013).

The present results suggest that GET73 induces a rightward shift of different glutamate receptor agonist concentration-response curves, possibly by acting as a NAM at mGluR5. Under some experimental conditions, the maximal effect of GET73 was observed in the nanomolar concentration range, thus suggesting a satisfactory potency of the compound in modulating mGluR5 signaling. However, the resulting potency of the compound, as well as its efficacy, differs among the different probe/assays. This is in line with the concept of GPCR-biased signaling, which has been also previously associated with mGluR5 allosteric modulation. For instance, the mGluR5 NAM M-5MPEP has been suggested to have differential negative cooperativity, partially inhibiting agonist-stimulated [^3^H]IP accumulation and completely blocking agonist-stimulated Ca^++^ oscillations (Bradley et al., 2011). Furthermore, the mGluR5 NAM VU0477573 only partially inhibited agonist-mediated intracellular Ca^++^ release and fully inhibited ERK_1/2_ phosphorylation (Nickols et al., 2016). These GPCR ligand features could also explain the observation that GET73 was able to shift the L-quisqualate CRC on pCREB and phosphatidylinositol turnover, but not on intracellular Ca^++^ levels. Finally, the present data indicate that the effects of GET73 were lower at higher than at lower concentrations. Although other possibilities cannot be ruled out, this observation could be in line with previous data suggesting that at higher concentrations GET73 might also exert a positive modulation at mGluR5 (Ferraro et al., 2011, 2013; Beggiato et al., 2013), thus dampening the negative effects observed at lower concentrations.

Another relevant finding of the present study is that the negative modulatory action of GET73 on mGluR5 ligand affinity is surmountable with respect to the agonist (i.e., the agonist produces the control maximal response). Thus, it seems likely that the compound could act as a surmountable allosteric modulator [i.e., allosteric modulators presenting a limiting value to the maximal displacement (Kenakin, 2009)]. However, further experiments are needed to confirm this hypothesis. Finally, the evidence that in the functional studies performed with different assays, GET73 (0.1 nM–10 μM) by itself did not exert any effects, indicates that the compound does not act as an agonist of mGluR5 or other glutamate receptor subtypes.

Although the *in vitro* preparations used in this study (i.e., cultured astrocytes or neurons, hippocampal slices) also express other glutamate receptor subtypes, the involvement of mGluR5 in the observed effects could be hypothesized. In fact, (i) GET73 negatively modulates the concentration-response curves of CHPG, which is a selective mGluR5 agonist (Doherty et al., 1997); (ii) all the analyzed intracellular pathways are associated with mGluR5 activation; (iii) previous studies demonstrated that GET73 does not act by modulating NMDA receptor function (Ferraro et al., 2011) and does not display affinity for ionotropic glutamate receptors (Loche et al., 2012).

The mGluR5 NAMs have reached or are now in active preclinical and clinical studies to evaluate the efficacy for several disorders, including alcohol use disorders (Ferraro et al., 2013; Mihov and Hasler, 2016). Despite encouraging results, both clinical and preclinical studies suggest that mGluR5 NAMs may induce on-target adverse effects, including cognitive and memory impairments and psychotomimetic effects (Campbell et al., 2004; Porter et al., 2005; Rodriguez et al., 2010; Abou Farha et al., 2014), raising the question of whether complete blockade of mGluR5 may contribute to these deleterious effects, thereby limiting the therapeutic utility of mGlu5 NAMs. On the other hand, according with Nickols et al. (2016) and Yang et al. (2016), a dramatic shift in the orthosteric agonist concentration-response curve is not necessarily a positive feature. For example, mGluR5 PAMs characterized by high potency in shifting the glutamate concentration-response curve, induce convulsions and neurotoxicity (Yang et al., 2016), while compounds possessing a lower efficacy show a safer profile, without losing *in vivo* behavioral activity. Analogous evidence indicates that full mGluR5 NAMs show a worse safety profile in respect to the partial NAMs (i.e., compounds that only partially block maximal mGlu5-mediated responses at concentrations that fully occupy the allosteric site on mGluR5; Nickols et al., 2016). Thus the properties displayed by GET73 in the present study, according with the recent literature, can be considered positive in terms of safety profile of the compound. In line with this view, no signs of neurotoxicity were observed in hippocampal neurons exposed to GET73 at concentrations ranging from 0.1 to 10 μM (Tomasini et al., 2016). More importantly, GET73 did not induce any CNS side effects in the two Phase I clinical trials completed so far in healthy volunteers (Haass-Koffler et al., 2017a; EudraCT registry number: 2011-002354-31), and in the ongoing Phase 1b/2a study in alcohol dependent subjects (Study 73CT203)^2^.

## Conclusion

Although further experiments are needed to explore GET73 binding at the orthosteric and/or allosteric sites on mGluR5, these data support the view that the compound acts as an mGluR5 NAM with a promising profile of action. Therefore, the present observations lend additional support to the significance of further investigating the possible mechanism of action of GET73.

## Author Contributions

SB, AB, MT, and NP contributed to calcium mobilization, phosphatidylinositol, and pCREB experiments, including the acquisition, analysis, and interpretation of data. LF participated in the above studies and drafted and revised the paper. MPC, AL, and RC contributed to revision of the paper.

## Conflict of Interest Statement

AL and RC are employees of Laboratorio Farmaceutico CT Srl, Sanremo, Italy. The other authors declare that the research was conducted in the absence of any commercial or financial relationships that could be construed as a potential conflict of interest.

## Footnotes

1 http://clinicaltrials.gov/ct2/show/NCT01842503

2 http://clinicaltrials.gov/ct2/show/NCT01842503

## ABBREVIATIONS

IP: inositol phosphate
NAM: negative allosteric modulator
PAM: positive allosteric modulator
pCREB: CREB phosphorylation.
