# Supplementary material for: In Vitro Functional Characterization of GET73 as Possible Negative Allosteric Modulator of Metabotropic Glutamate Receptor 5
Source: Front Pharmacol. 2018 Apr 5;9:327. doi: 10.3389/fphar.2018.00327 (PMC5895880; doi:10.3389/fphar.2018.00327)
Supplement: Supplementary file 3 [file Image_3.pdf]

frontiers

Supplementary Material

In Vitro Functional Characterization of GET73 as Possible Negative Allosteric Modulator of Metabotropic Glutamate Receptor 5

Beggiato Sarah, Andrea Celeste Borelli, Maria Cristina Tomasini, Maria Paola Castelli, Nicholas Pintori, Roberto Cacciaglia, Antonella Loche, Luca Ferraro*

*** Correspondence:** Luca Ferraro: frl@unife.it

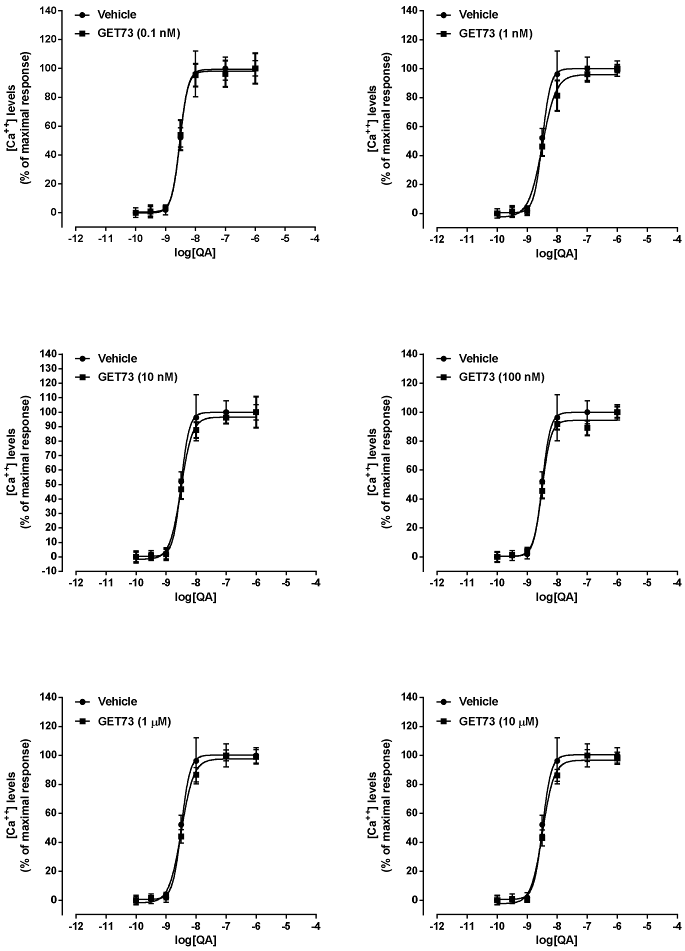

Supplementary Figure 3. Effects of GET73 (0.1 nM-10 μM) on L-quisqualate (QA) concentration-dependent increase in intracellular calcium levels in primary cultures of rat cortical astrocytes. The effects of the treatments on intracellular calcium levels are expressed as % of maximal response over the basal values. Each point represents the mean ± SEM (n=3). Concentration-response curves were generate using non-linear regression [curve fit, log(agonist) vs response, variable slope, four parameters].
